# Strong Reduction of Thermal Conductivity and Enhanced Thermoelectric Properties in CoSbS_1-x_Se_x_ Paracostibite

**DOI:** 10.1038/srep46630

**Published:** 2017-04-20

**Authors:** Radoslaw Chmielowski, Sandip Bhattacharya, Stéphane Jacob, Daniel Péré, Alain Jacob, Kenzo Moriya, Bruno Delatouche, Pascal Roussel, Georg Madsen, Gilles Dennler

**Affiliations:** 1 IMRA Europe S.A.S., 220 rue Albert Caquot, BP 213, 06904 Sophia Antipolis, France; 2 CMAT, ICAMS, Ruhr-Universität Bochum, Germany; 3 Unité de Catalyse et Chimie du Solide, Ecole Nationale Supérieure de Chimie de Lille, Bat C7a - BP 90108, 59652 Villeneuve d’Ascq, France; 4 Institute of Materials Chemistry, Technical University Vienna, A-1060 Wien, Austria

## Abstract

In order to reduce the thermal conductivity of CoSbS, a newly developed thermoelectric semiconductor, we have aimed at intentionally induce atomic disorder in its structure. This endeavor was guided by Density Functional Theory(DFT) calculations which indicated that substituting sulfur with selenium might be easily achievable experimentally because of the low formation energy of this point defect. Thereby, CoSbS_1−x_Se_x_ compounds having 0 ≤ *x* ≤ 1 have been synthesized by solid state reaction. Besides the expected semiconducting paracostibite phase, we have observed the appearance of a semimetallic costibite phase, never reported experimentally before. This cross-fertilized theoretical and experimental approach allowed us to reduce by 50% the thermal conductivity of paracostibite and therefore reach a maximum *z*T of 0.62 at 730 K. This makes this entirely new CoSbS_1−x_Se_x_ alloy very attractive for further optimizations and potential usage in thermoelectric applications.

Thermoelectrics generators(TEGs)^1^ have reached reasonably high Technological Readiness Level(TRL) since more than 160 years ago, when A. E. Bequerel *et al*. published the realization of a proof-of-concept, 30 leg TEG based on p-type Cu_2_S and n-type Cu-Ni-Zn alloy^2^. However, in spite of this early development, the market penetration of this renewable energy is still marginally small^3^. The main reasons for this situation, are(i) the low efficiency of TEGs,(ii) their manufacturing cost, and(iii) the cost of the thermoelectric(TE) materials they are based upon. One solution to the later issue consists of searching new TE materials, based on non-critical elements, and ideally naturally occurring as minerals. Tetrahedrite, which was recently shown to reach high TE performances is the archetype of such new materials^4^. However, tetrahedrite is a p-type semiconductor, and its n-type counterpart is still to be found. The performances of thermoelectric materials are characterized by the dimensionless figure of merit(*z*T) and power factor(PF) defined as *z*T = (S^2^ . σ/κ_total_)T and PF = S^2^ . σ, where S, σ, κ_total_ and T are the Seebeck coefficient, electrical conductivity, total thermal conductivity and temperature, respectively.

CoSbS paracostibite, a naturally occurring mineral, has recently been identified as an appealing n-type thermoelectric materials showing promising performances. In 2013, Parker *et al*. performed some Density Function Theory(DFT) calculations and highlighted a substantial band degeneracy near the conduction and valence band edges suggesting a large TE potential^5^. Doping CoSbS by substituting Co with Ni allowed the authors to reach a power factor of 1600 μW.K^−2^.m^−1^ and a *z*T of 0.35 at 773 K. This value of the traditional TE figure of merit was strongly limited by the high thermal conductivity of CoSbS, which was found to be about 7.5 and 3.5 W.K^−1^.m^−1^ at 300 K and 773 K, respectively. A couple of years later, Liu *et al*. also synthesized Co_1−x_Ni_x_SbS^6^ but contrarily to Parker *et al*. who used a vacuum melting approach, Liu *et al*. employed ball milling followed by hot pressing to prepare their samples. Interestingly, they could reach a maximum PF of 2000 μW.K^−2^.m^−1^ at about 800 K. Due to a lower thermal conductivity(5.5 W.K^−1^.m^−1^ at 300 K and 3.5 W.K^−1^.m^−1^ at 800 K) than the one reported by Parker *et al*., the highest *z*T value measured by Liu *et al*. was about 0.5 at 873 K. Very recently, Chmielowski *et al*. could show through DFT point defect calculations, that Te substitution on the Sb site should theoretically be a significantly more efficient dopant than Ni substitution on the Co site^7^. Experiments validated this conclusion: It was indeed observed that a 4% Te doping allowed to reach an unpresented PF of 2700 μW.K^−2^.m^−1^ at 543 K, which was maintained up to 730 K. To the best of our knowledge, this value is the highest reported for a polycrystalline chalcogenide material. Unfortunately, the thermal conductivity of this CoSb_1−x_Te_x_S was still found to be quite high(7 W.K^−1^.m^−1^ at 350 K and about 4 W.K^−1^.m^−1^ at 730 K). These values limited the best *z*T to 0.47 at 730 K.

The reduction of the thermal conductivity of a material without jeopardizing its electronic transport has become one of the major focus of the thermoelectric community during the last couple of decades. Various strategies have been proposed to elaborate *Electron Crystals Phonon Glasses*^8^ like phonon scattering^9^ at 2 of 3 dimensional interfaces, rattling atoms^8^, or atomic disorder^10^,^11^. In the case of metal sulfides or selenides, substituting partially sulfur ions by isoelectronic selenium ions(and vice-versa) has proved to be particularly efficient to reduce the thermal conductivity of materials like Cu_Y_Sb_Z_(S_1−x_Se_x_)^12^,^13^, while keeping their electronic transport properties unaltered.

Previous DFT calculations we had performed, indicated that the isoelectronic substitutional defect Se_S_ has a substantially low formation energy in paracostibite CoSbS(see Supporting information of ref. 7). This observation suggested that the sulfur - selenium alloying approach could, in principle, be implemented in order to increase notably its thermoelectric properties. Thus, we report herein the strategy we have employed in order to reduce by more than 50% the thermal conductivity of paracostibite. We show how cross-fertilization of ab-initio computations and experimental measurements allowed us, for the first time, to successfully synthesize two different phases of the mixed CoSbS_1−x_Se_x_ chalcogenide. High *z*T values above 0.6 at temperatures lower than 750 K could be achieved, what offers appealing perspectives for this new family of n-type sulfide thermoelectric material.

## Results

The first appearance of paracostibite(CoSbS) in the scientific literature dates back to 1970, when Cabri *et al*. reported the extraction and identification of this mineral from the Red Lake area in Ontario, Canada^14^. This phase shows an orthorhombic crystal structure(Pbca, 61), comprises 24 atoms in its unit cell, and possesses an indirect bandgap of about 0.4 eV.

Interestingly, another naturally occurring phase of CoSbS was identified by Cabri *et al*., namely costibite, discovered in ores extracted in Broken Hill, Australia^15^. Costibite is orthorhombic with space group Pnm2_1_(31) and comprises 6 atoms in its unit cell. Contrarily to paracostibite which was rapidly synthesized and reported to have a bandgap^16^, costibite could never be produced in laboratories and no electrical measurements have ever been reported on it.

In the case of CoSbSe, the scientific literature appears almost barren. We could only retrieve two fairly old publications reporting the synthesis and some shallow characterization of this material^16^,^17^. Both agreed on the crystalline structure of the compounds which was found to be orthorhombic with Pnn2 space group. Besides, Nahigian *et al*. reported a low resistivity of about 9 × 10^−4^ Ω.cm suggesting that this material may be of metallic nature^16^. As a matter of fact, while the two phases of CoSbS are present in several state-of-the-art ab-initio databases like the Materials Project^18^, the Open Quantum Materials Database^19^ or AflowLib^20^, CoSbSe remains absent from all of them, and has apparently never been computed.

### Theoretical results

**Figure 1:**
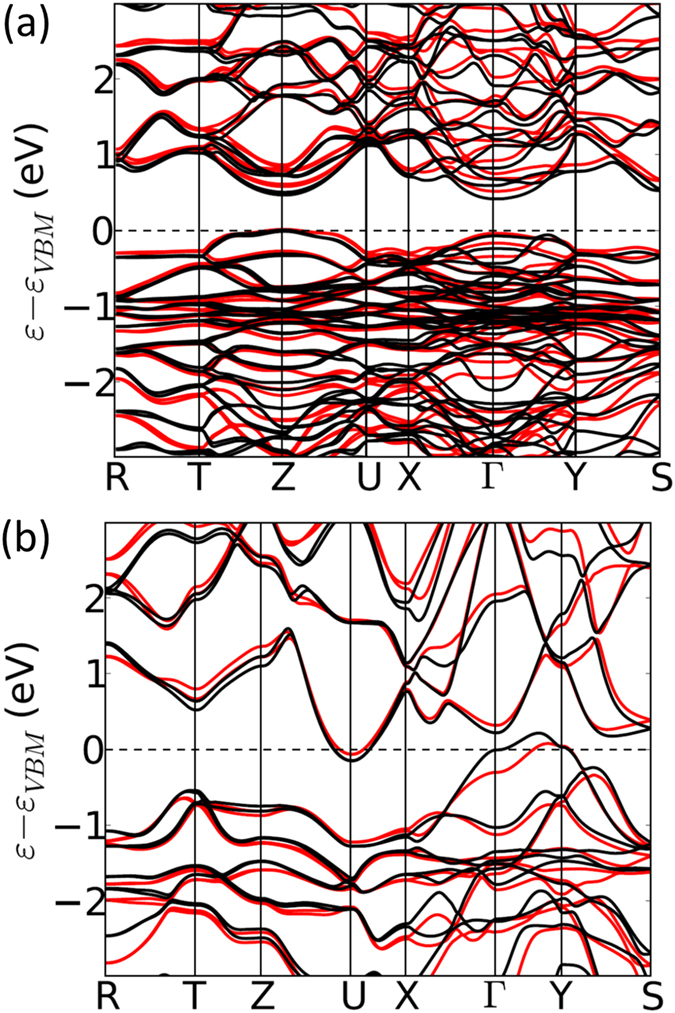
Calculated band structures. (**a**) Band structure of CoSbS paracostibite(Pbca, red line) and CoSbSe paracostibite(Pbca, black line)(**b**) Band structure of CoSbS costibite(Pnm2_1,_ red line) and CoSbSe(Pnm2_1,_ black line).

To clarify the relationship between CoSbS and CoSbSe phases, DFT calculations supported by experimental investigations have been performed in this work. Figure 1a, shows the band structures of the Pbca phase, CoSbS(in red) and the meta-stable CoSbSe(in black). These illustrate a semiconducting behavior with a finite bandgap, ε_g_(CoSbS) = 0.5 eV and ε_g_(CoSbSe) = 0.4 eV. Figure 1b shows the band structures of the Pnm2_1_ phase, metastable CoSbS(in red) and CoSbSe(in black), which illustrate a semi-metallic characteristic, with the U pocket of the Conduction Band Minimum(CBM) energetically below the Γ-Y pocket of the Valence Band Maximum(VBM). It should be noted that to perform these calculations, we have reexamined the crystal structure of CoSbSe and found the correct space group to be Pnm2_1_ as opposed to Pnn2 reported in the literature^16^,^17^. The X-ray diffraction patterns collected on synthesized materials of both CoSbS(Pbca) and CoSbSe(Pnm2_1_) as a function of temperature are presented in Supporting information in Figs S1 and S2, respectively. The atomic positions in the Pnm2_1_ phase were found by geometry optimization and are reported in the Supporting information, Table S1.

**Figure 2:**
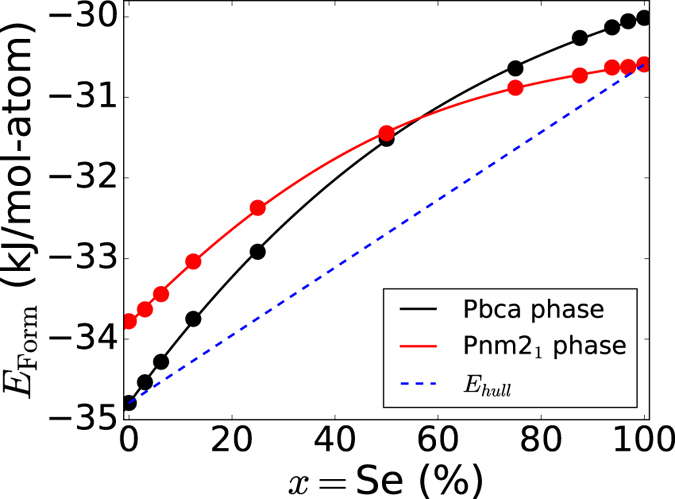
Energy of formation(in kJ/mol-atom) of CoSbS_1−x_Se_x_ paracostibite(Pbca) and costibite(Pnm2_1_) as a function of the alloying concentration, x.

We have then investigated theoretically the miscibility of CoSbS_1−x_Se_x_(Pbca) and CoSbS_1−x_Se_x_(Pnm2_1_) phases for alloy compositions of *x* = (3.125%, 6.25%, 12.5%, 25%, 50%, 75%, 87.5%, 93.75%, 96.875%), where *x* is the Se content. Figure 2, illustrates the energy of formation of CoSbS_1−x_Se_x_ alloys(Pbca in black, Pnm2_1_ in red). The convex hull of the system is a line connecting CoSbS(Pbca, *x* = 0) and CoSbSe(Pnm2_1_ *x* = 1) shown as dashed line in Fig. 2,


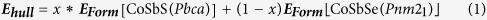


The mixing energy can be evaluated from the following equation:


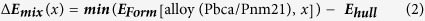


From Fig. 2, we observe that the mixing energy for any finite value of *x*(not 0, 100%), is less than 2 kJ/mol-atom. The low magnitude of mixing energy^21^ indicates that alloying the Sulphur position in CoSbS with selenium is conceivable. This would hold true despite the phase transition with increasing Se content in the alloy.

In order to understand the full alloy phase diagram, we have evaluated the Gibbs free energy using the following expression^21^,


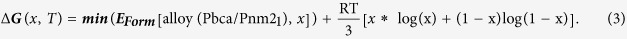


**Figure 3:**
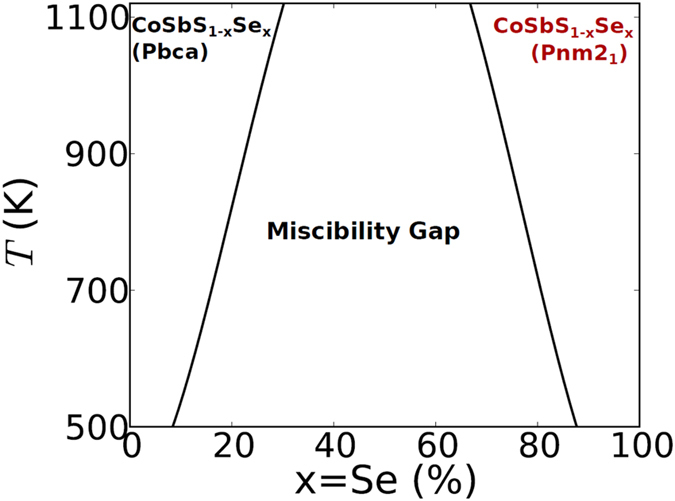
Temperature dependent phase diagram of CoSbS_1−x_Se_x_. Theoretically considerations indicate a presence of a miscibility gap. The single phase region is constituted by CoSbS_1−x_Se_x_(Pbca alloy) at low alloying concentrations and CoSbS_1−x_Se_x_(Pnm2_1_ alloy) at high alloying concentrations.

Here the last term accounts for the configurational entropy in the alloy. At each temperature, this gives us a *x*(Se content) dependent function: i.e. *ΔG(x, T*). For a pseudo-binary alloy of CoSbS_1−x_Se_x_, we obtain a mechanical mixture at T = 0 K, where the entropy term in Eq.(3) reduces to zero. At very high temperatures, Eq.(3) will only be governed by the entropy term, which will give a single minimum in the *ΔG(x*) curve. However at intermediate temperatures, the opposing contribution to *ΔG(x*) from the enthalpy and entropy terms gives two minima at points *x*_*1*_(Se poor) and *x*_*2*_(Se rich) and the free energy is minimized by a two-phase mixture. The boundary of the two-phase region is obtained by constructing the common tangent in the free-energy curve. This analysis gives us the temperature dependent phase diagram with a miscibility gap, as shown in Fig. 3.

The soluble region of the phase diagram consists of CoSbS_1−x_Se_x_(Pbca alloy) for low alloying(Se poor) concentrations and CoSbS_1−x_Se_x_(Pnm2_1_ alloy) for high alloying(Se rich) concentrations. Taking a growth temperature at about 800 K we would expect a mixture of a S rich Pbca phase and a Se rich Pnm2_1_ phase for 0.2 < *x* < 0.8. However, it should be noted that the theoretical phase diagram somewhat underestimates the miscibility regions as the vibrational entropy which generally stabilizes the alloy phases is not taken into account^22^.

### Experimental results

**Figure 4:**
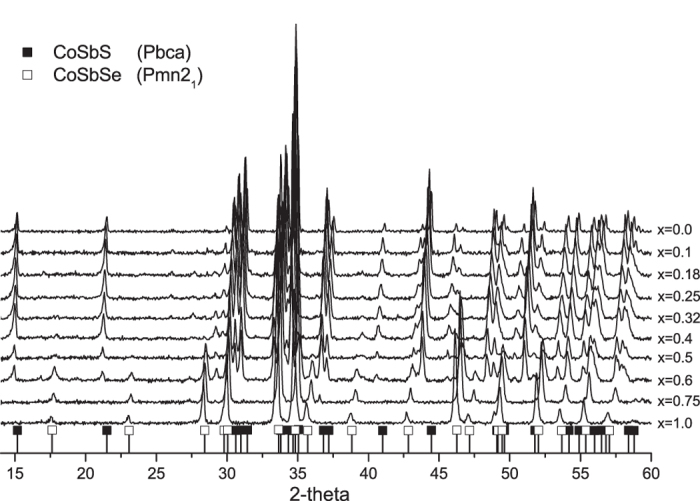
X-ray diffractions patterns collected at room temperature on CoSbS_1−x_Se_x_ alloys. X is varying from 0 to 1. Only peaks with relative intensity above 10% are shown.

In order to investigate and comprehend the influence of the content of Se on the thermoelectric properties in CoSbS_1−x_Se_x_ alloy, we have synthesized a series of 10 samples with different *x* values, covering the whole range going from 0 to 1. The X-ray diffraction(XRD) patterns of the various samples, recorded at room temperature are shown in Fig. 4. As expected for *x* = 0, the pattern obtained in this case corresponds to the paracostibite(Pbca) phase, reported earlier^7^. However, in the case when *x* = 1, the crystal structure is clearly of a different nature. A comparison between the XRD pattern collected for *x* = 1 and both Pnn2 and Pnm2_1_ symmetries is shown in Fig. S3. The absence of two XRD peaks in the Pnn2 fitting clearly indicates that CoSbSe crystalizes in Pnm2_1_ symmetry and not like previously reported in Pnn2. To the best of our knowledge, our study makes the first appearance in the scientific literature of the full diffractogram of CoSbSe. This particular value of *x* was then thoroughly recorded from 2θ = 12 to 130° and refined using the Rietveld approach with results given in Fig. S4 and Table S2 in the Supporting information.

When *x* increases from 0 on, all the peaks of paracostibite shift to smaller angles. This clearly indicates that the interplanar distances within CoSbS Pbca lattice are rising. The fact that this shift appears smooth without the appearance of different population(i.e. CoSbS + CoSbS_1−x_Se_x_) tends to indicate that, as suggested by our DFT calculations presented above, Se is easily miscible in the CoSbS phase. This situation resembles somewhat the one observed recently in the case of Cu_2_ZnSnS_4−x_Se_x_, a novel material used for photovoltaic applications^23^. Thus, we have interpreted this tendency as a steric effect, i.e. an increasing substitution of small S atoms by bigger Se atoms. Interestingly, the opposite tendency can be noted when *x* is decreased from 1 on. Indeed, it appears that the peaks attributed to the CoSbSe Pnm2_1_ phase shift toward larger angles as *x* decreases. We interpret this phenomenon as a substitution of Se by S within the Pnm2_1_ CoSbSe phase. Finally, the collected diffractograms show that for 0.4 ≤ *x* ≤ 0.6 both paracostibite(Pbca) and costibite(Pnm2_1_) CoSbS_1−x_Se_x_ alloys coexist. To confirm these tendencies we have performed LeBail refinements(full pattern matching) of the diffractograms presented in Fig. 4.

**Figure 5:**
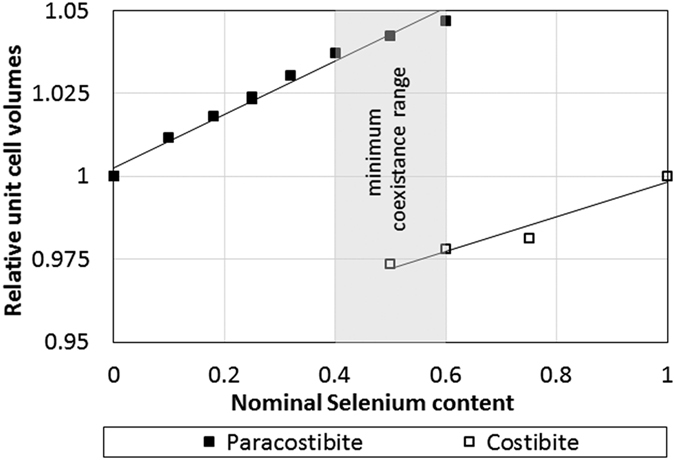
Evolution of the relative unit cell volumes V/V_0_ as a function of nominal selenium content. The minimum coexistence range from XRD patterns is 0.4 ≤ x ≤ 0.6. For x = 0.4 the costibite is detected, however the intensity of the XRD peaks is too low to perform a LeBail refinement.

However, as the unit cell parameters of paracostibite and costibite phases are strongly different, the comparison was performed using the relative volume, allowing an easier distinction between them. The relative volume is defined as: V_x_/V_0_ where V_0_ is the unit cell of the non-alloyed CoSbS and CoSbSe phases and V_x_ the volume defined from the LeBail refinement for each selenium concentration. Figure 5 shows the variation of the relative volumes as a function of the nominal selenium concentration for both paracostibite and costibite phases. It clearly appears that alloying CoSbS with selenium causes an important increase of the relative volume of paracostibite: For instance, an alloying with *x* = 0.4 increases the unit cell by 3.7% in comparison to the pure phase. In the case of the costibite phase, an inverse trend is observed. Alloying with *x* = 0.6 causes a decrease by about 2.2% in comparison to pure costibite phase. The comparison of the slopes indicates that alloying with selenium has a much stronger impact on the volume of the paracostibite phase than on the volume of costibite.

**Figure 6:**
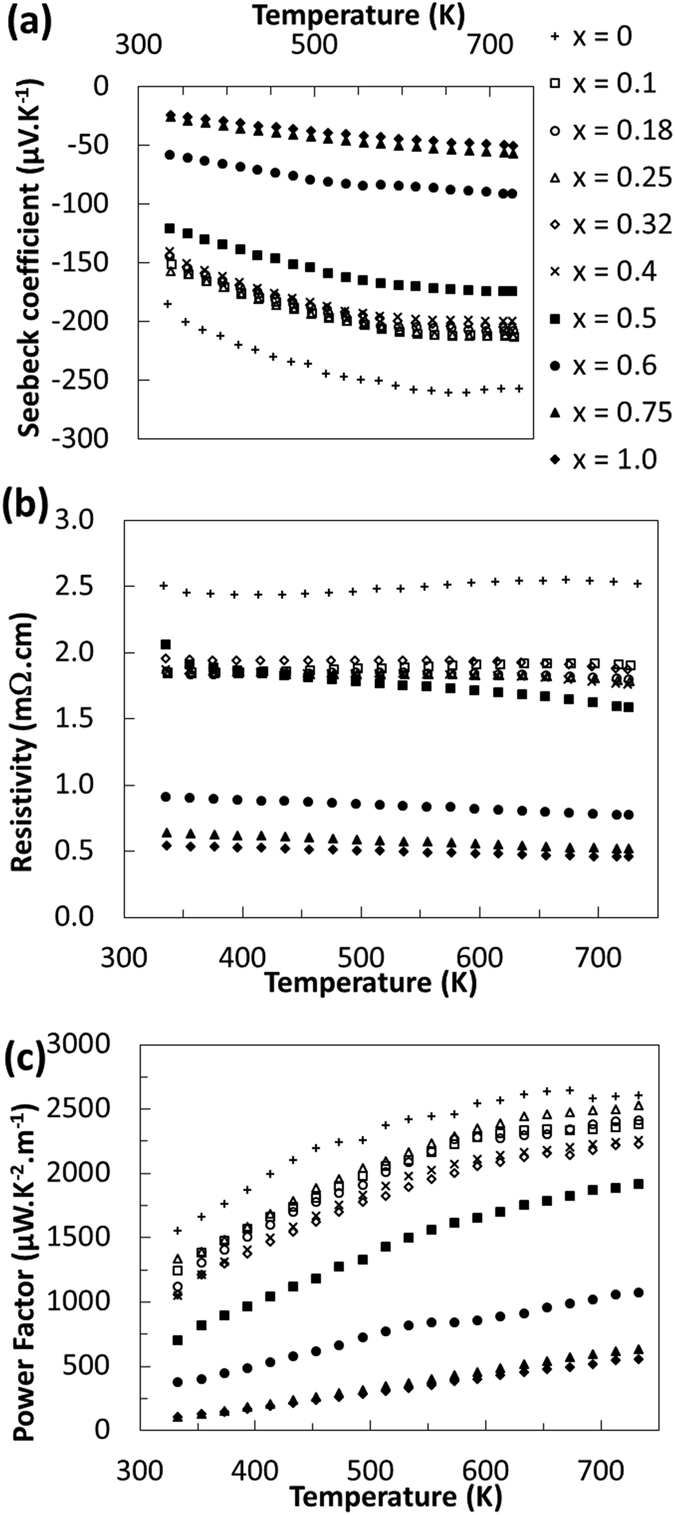
Electronic transport properties of CoSbS_1−x_Se_x_ doped with Te. (**a**) Seebeck coefficient,(**b**) electrical conductivity, and(**c**) Power Factor as a function of x and the temperature of measurement.

To evaluate the effect of sulfur - selenium alloying on the thermoelectric properties of these compounds, temperature dependent Seebeck coefficient and electrical resistivity measurements have been carried out. The summary of these transport properties as well as the resulting power factor are presented in Fig. 6. The variations of the electronic transport properties are in a good agreement with the crystal structure variation observed by X-ray diffractions. As one can see, when paracostibite is the major phase, high thermoelectric performances due to the high absolute Seebeck coefficient are observed. We believe that in the range of 0 < *x* < 0.6 the semiconducting nature of paracostibite is driving the thermoelectric properties of the material. It is worth mentioning here that the electronic transport properties are not strongly affected by the selenium substitution as this substitution is isoelectronic. In contrary, when costibite phase is the major phase in the range of 0.6 ≤ *x* ≤ 1 the absolute Seebeck coefficient as well as the electrical resistivity are significantly lower and the resulting power factor is low. We believe that these modifications affecting the thermoelectric performances are due to the semimetallic behavior of the costibite phase.

**Figure 7:**
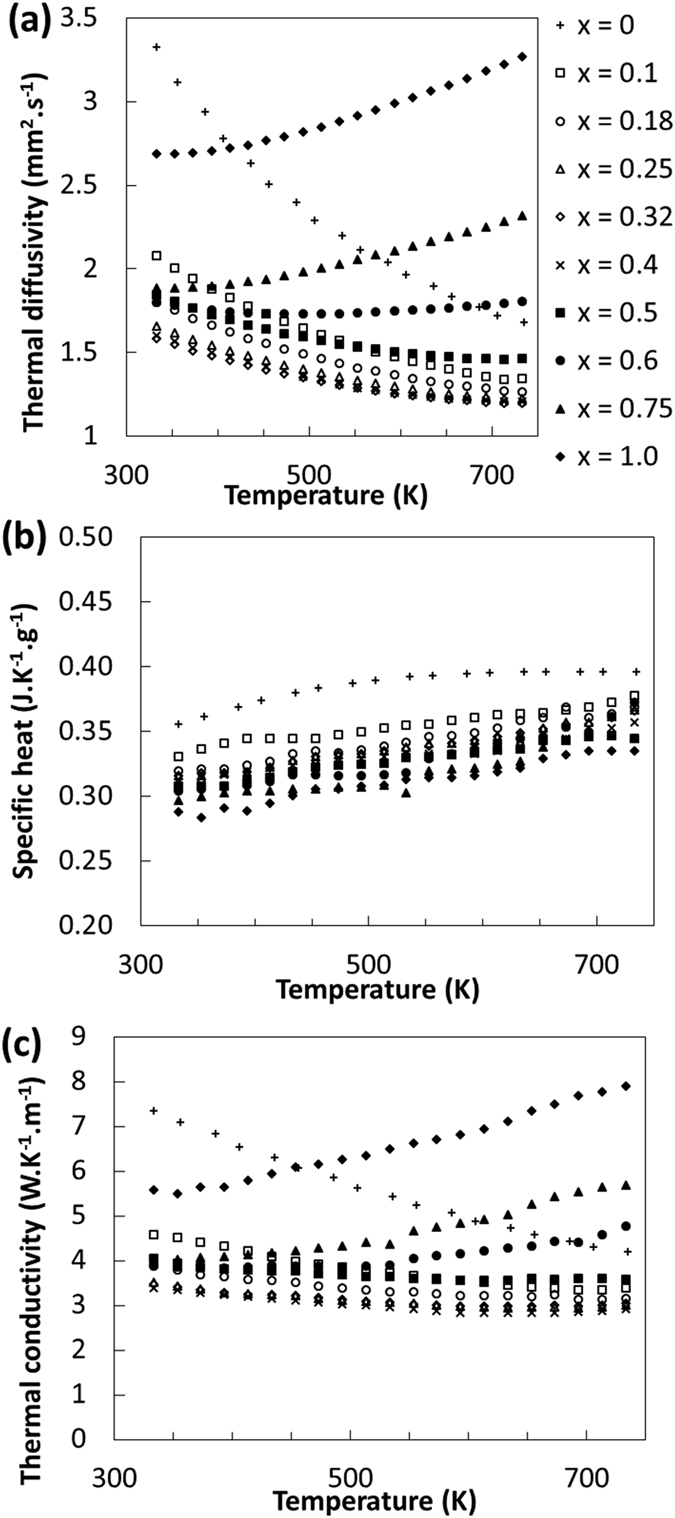
Thermal transport properties of CoSbS_1−x_Se_x_ doped with Te. (**a**) Thermal diffusivity,(**b**) Heat capacity, and(**c**) Total thermal conductivity as a function of x and the temperature of measurement.

To assess the overall effect of the partial sulfur - selenium substitution on the thermal properties, thermal diffusivity and heat capacity measurements have been performed. As one can see in Fig. 7, the variations of the heat capacity are nearly negligible in comparison to the variations of the thermal diffusivity. Indeed, the thermal diffusivity of the semiconducting paracostibite phase(*x* = 0) decreases from about 3.5 mm^2^.s^−1^ at room temperature to about 1.65 mm^2^.s^−1^ at 733 K. An opposite trend is observed for the semimetallic costibite phase(*x* = 1) for which the thermal diffusivity rises from 2.7 mm^2^.s^−1^ at room temperature to 3.5 mm^2^.s^−1^ at 733 K. Surprisingly, for all alloys, the thermal diffusivity is lower than the one observed for both pure paracostibite and costibite phases, on nearly the entire temperature range. Finally, the thermal diffusivity of alloys is about 2 mm^2^.s^−1^ at room temperature and decreases to 1.3 mm^2^.s^−1^ at 733 K, i.e. lower than the value observed for both paracostibite and costibite phases. As there are nearly no effects on the heat capacity, the resulting total thermal conductivity is driven by the trends observed in thermal diffusivity.

**Figure 8:**
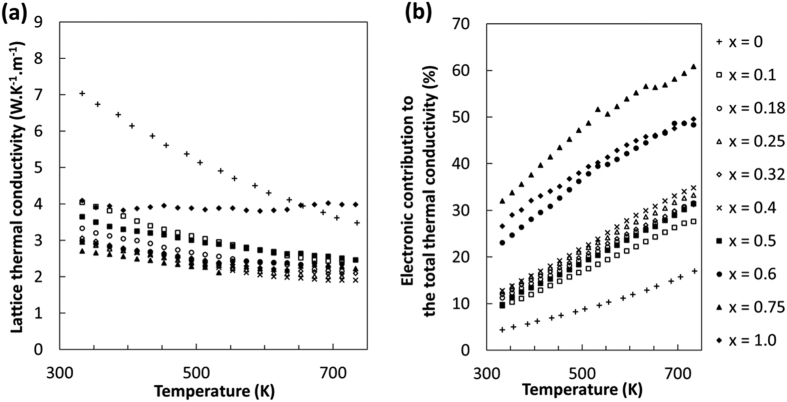
Thermal transport properties of CoSbS_1−x_Se_x_ doped with Te. (**a**) Lattice thermal conductivity as a function of temperature,(**b**) electronic contribution of the total thermal conductivity as a function of temperature.

To evaluate the contribution of the electronic(κ_e_) and lattice(κ_l_) parts of the total thermal conductivity(κ_total_), the electronic part of the thermal conductivity has been calculated using the Wiedemann-Franz relation, κ_e_ = L.σ.T, where L is the Lorentz number(2.44 × 10^−8^ W.Ω.K^−2^), σ is the electrical conductivity, and T is the temperature. The κ_total_ is assumed to be a sum of κ_e_ and κ_l_. As one can see in Fig. 8a, the lattice thermal conductivity of CoSbS_1−x_Se_x_ when *x* = 0 is relatively high but decreases from 7.03 at 333 K to 3.48 W.K^−1^.m^−1^ at 733 K with an approximately T^−1^ dependence. This tends to indicate that the phonon-phonon scattering dominates the phonon transport. The alloying with selenium leads to a considerable decrease of the κ_l_ contribution as for *x* = 0.25 the κ_l_ is only 3.07 and 2 W.K^−1^.m^−1^ at 333 K and 733 K, respectively. For the *x* = 0.25 and *x* = 0.4 samples, the mass variance parameter^24^ ranges approximately from 0.25 to 0.5 for the mixed S/Se site. This suggests that almost a third of the thermal conductivity may be suppressed solely due to mass disorder scattering. Furthermore, for *x* = 0.4, the average mass of the unit cell will increase by 10%. As expected, the combination of these two effects can explain the drop in lattice thermal conductivity from 5 to 3 W.K^−1^.m^−1^ at the peak performance temperature.

**Figure 9:**
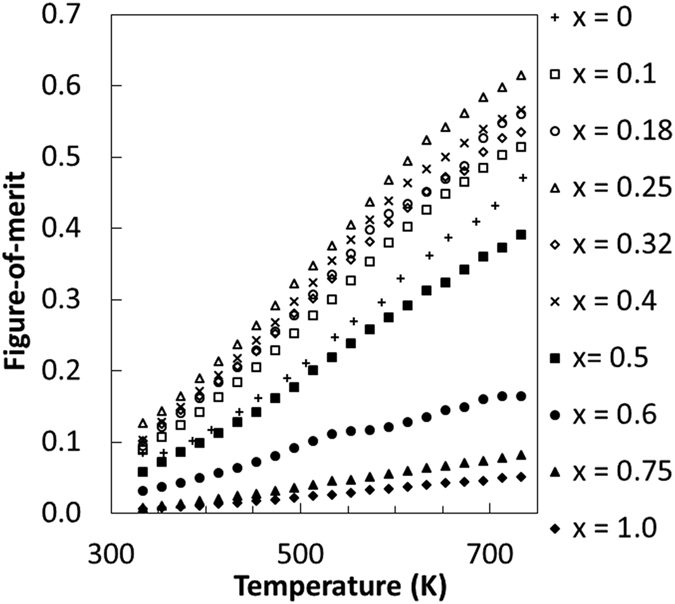
Figure of merit *z*T of CoSbS_1−x_Se_x_ doped with Te as a function of *x* and the temperature of measurement.

Finally, all alloy compositions in the range of 0 < *x* ≤ 0.4 exhibit higher thermoelectric figure of merit *z*T than the one observed at *x* = 0(Fig. 9). A maximum *z*T of 0.62 has been obtained at 733 K for 25% substitution of sulfur by selenium.

**Figure 10:**
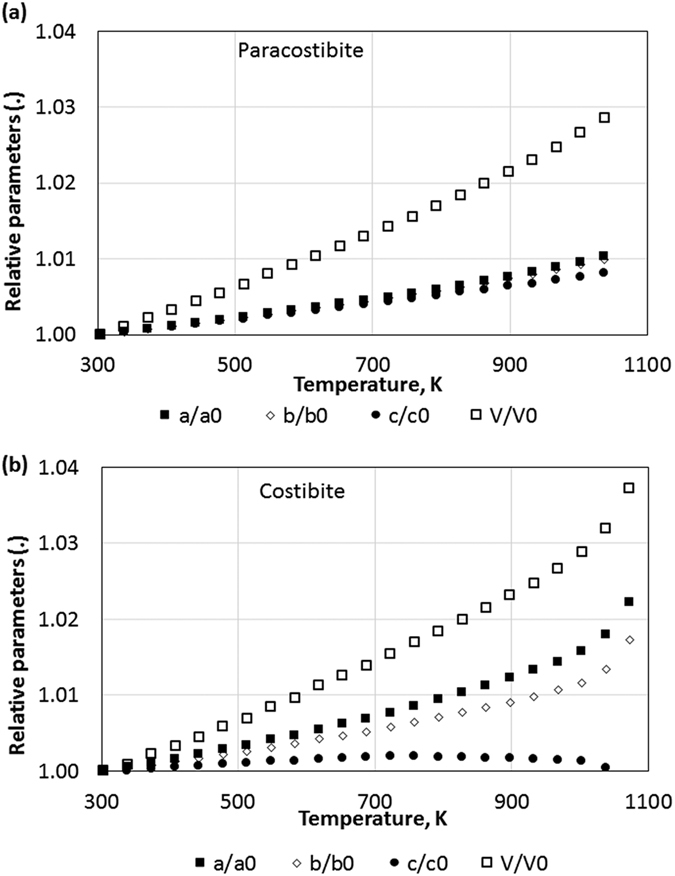
Variation of the relative parameters a/a_0_, b/b_0_, c/c_0_ and V/V_0_ as a function of temperature for(**a**) paracostibite,(**b**) costibite.

The temperature dependent X-ray diffraction measurements performed at *x* = 0 and *x* = 1(Figs S1 and S2) allow us to access to the information about thermal expansion and stability of these compounds. The variation of the relative parameters as a function of temperature is presented on Fig. 10. In the case of the paracostibite phase, one can see that all relative unit cell parameters show a linear isotropic expansion. The estimated total volume increase is about 2% at 873 K, compared to the value at room temperature. In the case of costibite phase, the relative *a* and *b* unit cell parameters expand in an identical manner while the relative *c* parameter is nearly constant. This clearly indicates an anisotropic expansion of the costibite lattice. It is worth to mention here that we did not observe any decomposition of CoSbS(Pbca) up to 1023 K while the CoSbSe(Pnm2_1_) starts to decompose at about 950 K.

## Discussion

The experimental measurements reported above agree well with the low formation energy of the Se_S_ point defect in paracostibite^7^. By simply changing the stoichiometry of the starting materials and keeping all the other experimental parameters unchanged, we could substitute sulfur by selenium on the whole range going from 0 to 100%. No secondary phases could be identified, but a phase change from paracostibite to costibite appears with increasing selenium content, exactly as forecast by the DFT results shown in Fig. 2. This phase change upon *x* increase makes the case of CoSbS different from Cu_2_ZnSnS_4−x_Se_x_ which maintains the same kesterite structure for all values of *x*^25^.

Figure 4, furthermore indicates that semimetallic costibite is the sole structure present in the alloy for *x* ≥ 0.6. Therefore, we attribute the decrease of the Seebeck coefficient upon increasing x for *x* ≥ 0.6, to the penetration of the Fermi level into the bands. Indeed, as exhibited in Fig. 1, CoSbSe costibite has a deeper valley at U point and higher hill at Y point than CoSbS costibite has. This larger overlap of the integrated density of state induces an increase of the number of carriers, as well as a limited change of carrier velocity upon increasing temperature(Seebeck coefficient).

In the range 0.4 ≤ *x* ≤ 0.6, when both paracostibite and costibite phases co-exist, we can observe an evolution from a metallic behavior(for *x* = 0.6) to a semiconducting behavior(for *x* = 0.4). This indicates that within this composition range, the thermoelectric properties of CoSbS_1−x_Se_x_ is driven either by the paracostibite or the costibite phase.

Finally, the most interesting range(of *x)* for thermoelectric applications seems clearly to be 0 ≤ *x* < 0.4, in which only the semiconducting paracostibite phase is present. In this range a simultaneous decrease in the absolute Seebeck coefficient and electrical resistivity with increasing *x* is observed. We attribute this effect to an increase of the number of charge carriers in the materials. This increase can have several sources. One may invoke a decrease of the band-gap of the alloy as *x* increases. However, Fig. 1 suggests that this effect may be quite negligible: Indeed, assuming that CoSbS paracostibite has a band-gap of 0.6 eV and that its CoSbSe counterpart has band-gap of 0.5 eV, a linear regression between these two values would conclude that CoSbS_0.9_Se_0.1_ should have a band-gap of 0.59 eV. This small variation is obviously not sufficient to explain a decrease of 20% of the resistivity and the Seebeck coefficient as observed on Fig. 6 between the two cases *x* = 0 and *x* = 0.1. We think that a small fraction of Se atoms may enter the Sb site, so as to create a slight increase in carrier concentration. Indeed, our previous DFT calculations have indicated that while Te_Sb_ is the substitutional defect having the lower formation energy(0.2 eV at the VBM in Co rich condition), Se_Sb_ is quite probable too(0.6 eV at the VBM in Co rich condition)^7^. This was confirmed by experimental results showing that Se can be used as a n-dopant, even though it remains less efficient than Te(10^20^ cm^−3^ carriers with 2% Te doping versus 10^19^ cm^−3^ carriers with 2% Se doping, the undoped materials showing a carrier density of 5 × 10^18^ cm^−3^). Thus it is likely that when increasing *x* from 0 to 0.1, the carrier concentration also increases by a small fraction due to some Se_Sb_ substitution. This would explain the decrease in both Seebeck coefficient and resistivity, between *x* = 0 and *x* = 0.1. As *x* increases further, this effect saturates swiftly, and both the Seebeck coefficient and the electrical resistivity remain almost unchanged(till *x* = 0.4), until the costibite phase starts to appear in the material.

As a conclusion, we have demonstrated throughout this study that the sulfur - selenium alloying is an efficient way to decrease the total thermal conductivity in the CoSbS - CoSbSe system. This alloying may causes an important atomic disorder on the chalcogenide sites which in turn may induce an efficient phonon scattering mechanism. On the other hand, as S and Se are isoelectronic, this alloying has only a limited impact on the electron transport properties, beside the small appearance of Se_Sb_ n-doping point defects. With this approach, we could induce an enhancement of about 35% of the thermoelectric performances(*zT*) which suggests a strong future potential for this class of compounds.

Finally, we would like to underline that this study clearly illustrates how cross fertilization between theoretical computation and experimental measurement can increase the understanding of complex materials systems, and accelerate the identification and the optimization of compounds pertinent for dedicated applications.

## Methods

### Calculation Methods

The Crystallographic Information File(CIF) for CoSbS(Pbca) and CoSbS(Pnm2_1_) phases that are known experimentally were extracted from the Pearson’s database^26^. We then relaxed the volume of both structures till the forces on each atom were less than 0.01 eV/Å and the stress in the cell is negligible. DFT structural relaxation was performed using the VASP code with projected augmented wave method with a cut-off of 500 eV and k-mesh of 10 × 10 × 5(Pbca phase) and 16 × 12 × 10(Pnm2_1_ phase). The resultant DFT total energy, atomic positions and cell volumes for both relaxed structures agree quite well with the Materials Project database^18^. Thereafter, all the S atoms in the CoSbS(Pbca) unit cell were replaced with Se to obtain the starting CoSbSe(Pbca) structure. Likewise, all the S atoms in the CoSbS(Pnm2_1_) were replaced with Se to obtain the starting CoSbSe(Pnm2_1_) structure. The volume of the starting structures of both the selenide compounds were then relaxed by finding the minimum in their respective energy volume curves. Furthermore, for both phases the atomic coordinates were relaxed till the forces on each atom were less than 0.01 eV/Å and the stress in the cell is negligible.

Comparing the DFT formation energies for the relaxed CoSbS compositions, we obtain that the Pbca phase lies on the convex hull, while the Pnm2_1_ phase is 10.5 meV/atom above the stable phase. This is in good agreement with the formation energy values for both phases in the Materials Project database. Interestingly, however for the CoSbSe we observe the opposite, i.e. the Pnm2_1_ phase is on the convex hull, while the Pbca phase is 6.0 meV/atom above the stable phase.

We then performed self-consistent DFT calculations using the(L)APW+lo method^27^ implemented in the WIEN2k code^28^ with the relaxed structures of all four compounds. These were followed by band structure calculations on a fine k-mesh of 64 × 10^6^/V_unitcell. DFT band structures were evaluated using Engel-Vosko(EV) functional^29^, which were demonstrated to estimate the bandgaps quite accurately(compared to standard LDA/GGA) for thermoelectric materials with very low computational costs^30^.

For both phases the structures of the intermediate alloy compositions were generated using Special Quasirandom Structures(SQS)^21^,^31^ using the ATAT code^32^, on a 2 × 2 × 1 super-cell for the Pbca phase and 4 × 2 × 2 super-cell for Pnm2_1_. DFT total energy calculations were done on these SQS alloy structures with plane-wave energy cut-off of 500 eV.

### Samples synthesis and characterizations

Tellurium doped CoSbS_1−x_Se_x_ alloys where x = 0, 0.1, 0.18, 0.25, 0.32, 0.4, 0.5, 0.6, 0.75, and 1.0 were prepared using the same procedure as described in our previous work^7^. The raw powders of Co, Sb, Te, from Goodfellow, S from Sigma-Aldrich and Se from Alfa Aesar were mixed with the required amount.

Electrical resistivity and Seebeck coefficient were measured under helium atmosphere from 340 K to 733 K on a LSR-3 setup from Linseis. The thermal conductivity(*κ*) was calculated via *κ* = *D*_*f*_ . *C*_*p*_ . *d*, where *D*_*f*_ is the thermal diffusivity, *C*_*p*_ is the heat capacity and *d* is the density. The thermal diffusivity was measured by the xenon flash method on a Netzsch LFA 467 under nitrogen flow and using Cape-Lehman fitting. The thermal capacity measurements were carried out on a PerkinElmer DSC8000 with a Pt-Ir/Al crucible and a ramping rate of 20K.min^−1^. The X-ray diffraction(XRD) patterns were collected at room temperature on powders using a D8 Bruker X-ray diffractometer equipped with a copper anode and a point detector. The *in-situ* high temperatures XRD patterns were collected from 303 K up to 1038 K with temperature steps of 35 K using a Bruker D8 Advance diffractometer equipped with a LynxEye 1D detector and a high temperature furnace(Anton Paar XRK900) equipped with a Macor© glass ceramic sample holder. The Jana2006 software^33^, option powder, was used for the structural refinements(Le Bail fitting for HTXRD study and Rietveld approach for the *x* = 1 compound) using a Pseudo-Voigt profile function and a manually pointed background convoluted with a 10 terms Legendre polynomial.

## Additional Information

**How to cite this article**: Chmielowski, R. *et al*. Strong Reduction of Thermal Conductivity and Enhanced Thermoelectric Properties in CoSbS_1-x_Se_x_ Paracostibite. *Sci. Rep.* **7**, 46630; doi: 10.1038/srep46630(2017).

**Publisher's note:** Springer Nature remains neutral with regard to jurisdictional claims in published maps and institutional affiliations.

## Supplementary Material

Supplementary Information
