# Supplementary material for: Strong Reduction of Thermal Conductivity and Enhanced Thermoelectric Properties in CoSbS1-xSex Paracostibite
Source: Sci Rep. 2017 Apr 20;7:46630. doi: 10.1038/srep46630 (PMC5397971; doi:10.1038/srep46630)
Supplement: Supplementary Information [file srep46630-s1.pdf]

Supporting Information

Strong Reduction of Thermal Conductivity and Enhanced Thermoelectric Properties in $CoSbS_{1-x}Se_x$ Paracostibite

Radoslaw Chmielowski[a], Sandip Bhattacharya[b], Stéphane Jacob[a], Daniel Péré[a], Alain Jacob[a], Kenzo Moriya[a], Bruno Delatouche[a], Pascal Roussel[c], Georg Madsen[d], and Gilles Dennler[a]

[a)]IMRA Europe S.A.S., 220 rue Albert Caquot, BP 213, 06904 Sophia Antipolis, France.
[b)]CMAT, ICAMS, Ruhr-Universität Bochum, Germany.
[c)]Unité de Catalyse et Chimie du Solide, Ecole Nationale Supérieure de Chimie de Lille, Bat C7a - BP 90108, 59652 Villeneuve d'Ascq, France.
[d)]Institute of Materials Chemistry, Technical University Vienna, A-1060 Wien, Austria.

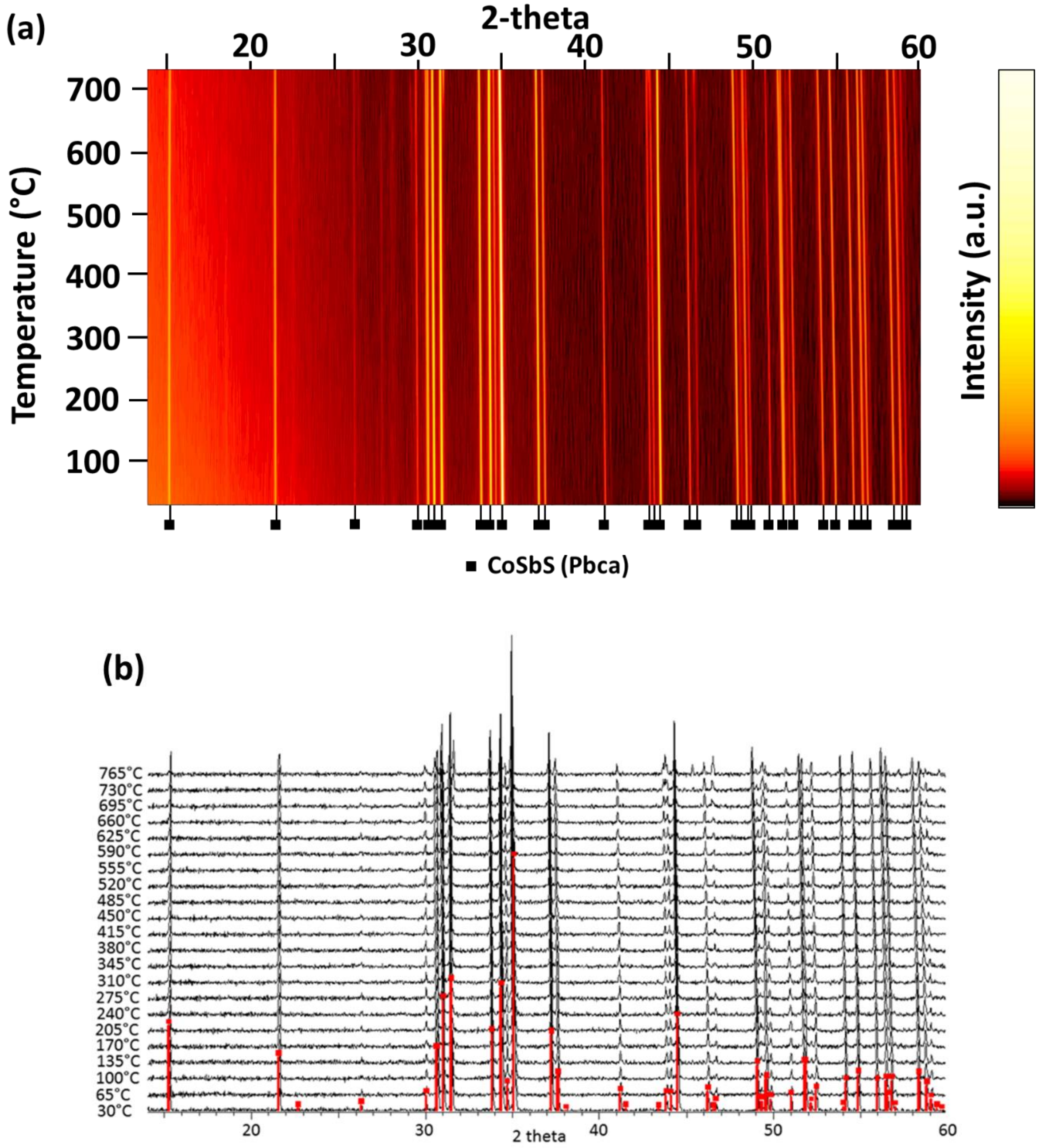

Figure S1. (a) Isodensity map reconstructed from all *in-situ* temperature dependence XRD patterns collected on 4at% Tellurium doped CoSbS. (b) Same data presented as a series of temperature dependence XRD patterns. The fitting of the unit cell parameters has been performed using Pbca space group as reference.

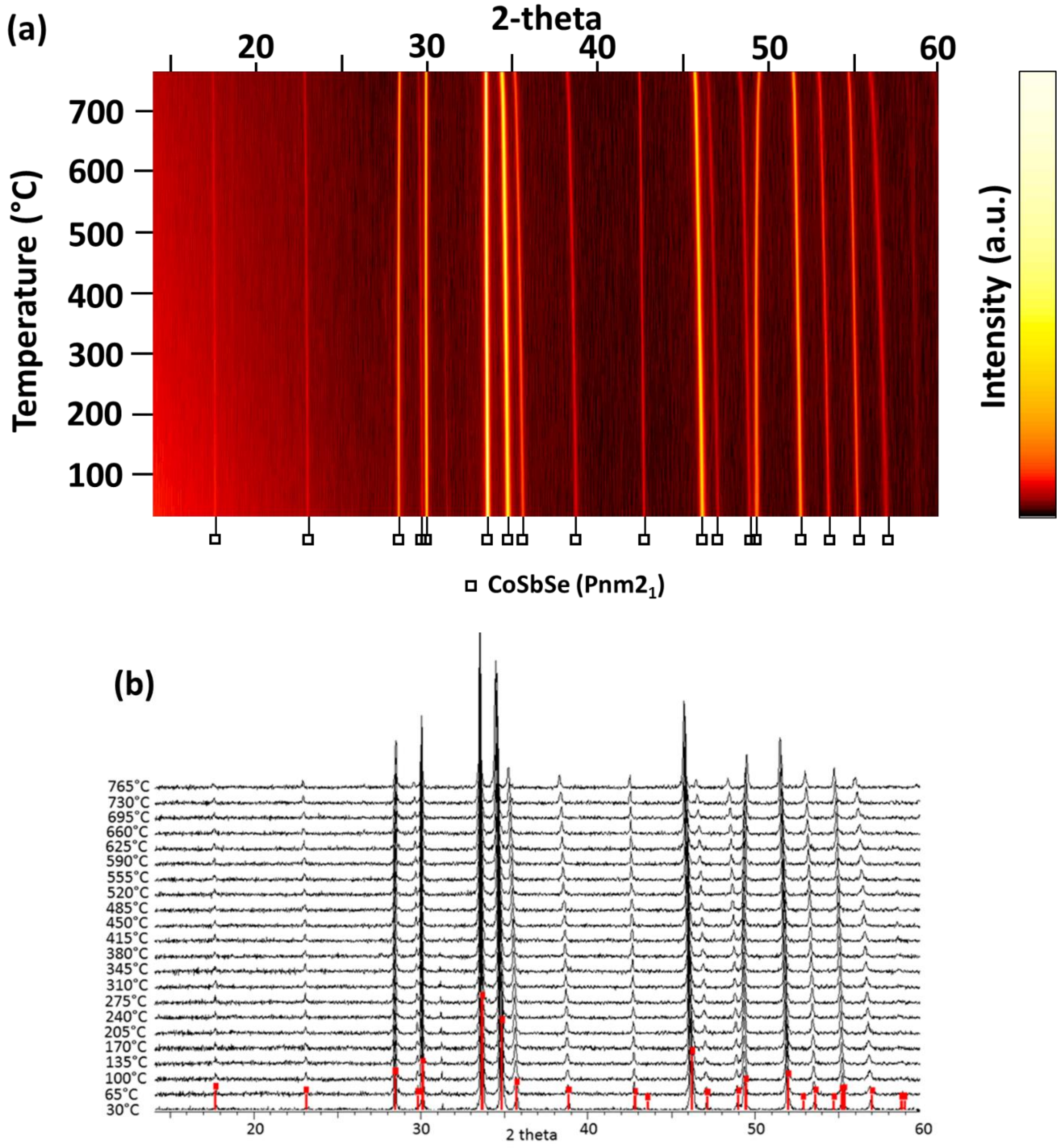

Figure S2. Isodensity map reconstructed from all *in-situ* temperature dependence XRD patterns collected on CoSbSe. (b) Same data presented as a series of temperature dependence XRD patterns. The fitting of the unit cell parameters has been performed using Pnm2$_1$ space group as reference.

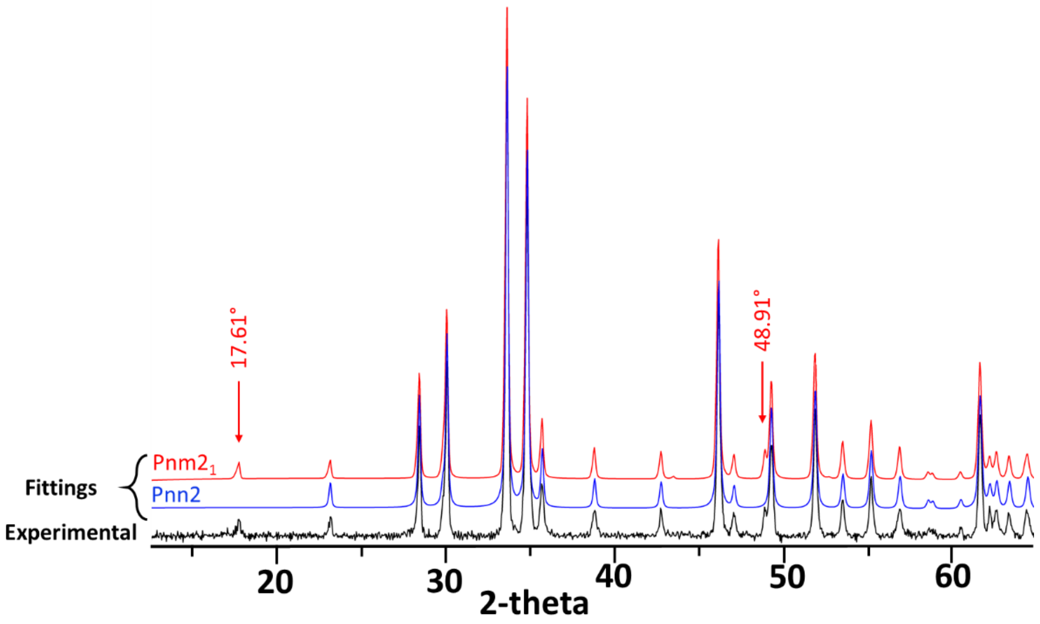

Figure S3. Comparison of the CoSbSe XRD patterns with $Pnm2_1$ and Pnn2 fittings. The absence of two peaks at 2theta 17.61° and 48.91° on the Pnn2 fitting clearly indicates that CoSbSe crystal structure fits better with $Pnm2_1$.

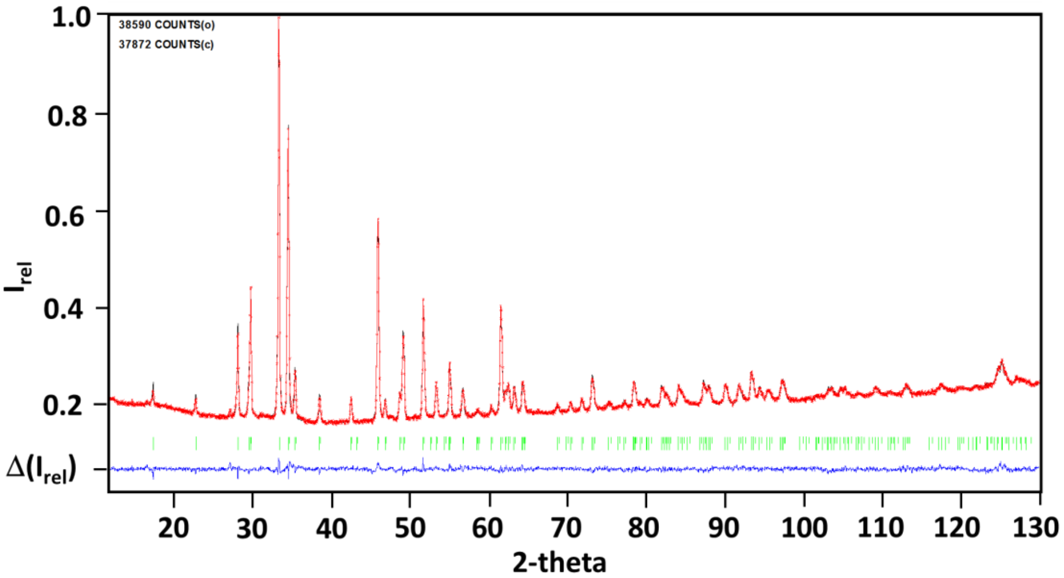

Figure S4. The Rietveld refinement performed on X-ray powder diffraction data collected on pure CoSbSe at RT. Sticks indicate Bragg peak positions. The difference between the experimental and calculated data is given underneath. The agreement factors are given in Table S2.

Table S1. Experimental (Rietveld refinement) and calculated unit cell parameters of CoSbSe ($Pnm2_1$). Optimized atomic positions of Co, Sb and Se obtained using the PBE functional.

Experimental	a (pm)	b (pm)	c (pm)
	369.25(2)	503.73(3)	600.94(3)
	x	y	z
Sb	0.5	0.464(2)	0.4978(6)
Co	0.5	0.223(2)	0.869(3)
Se	0.5	0.039(2)	0.2305(7)

Calculated	a (pm)	b (pm)	c (pm)
	371.6	501.9	602.8
	x	y	z
Sb	0.5	0.471	0.5
Co	0.5	0.231	0.865
Se	0.5	0.037	0.228

Table S2. The agreement factors of the Rietveld refinement performed on CoSbSe ($Pnm2_1$) presented on Fig. S4.

Profile parameters

R_p	wR_p	Number of points	Number of parameters	Npnts/Np	Goodness of Fit
1.22	1.57	2951	39	75.67	1.42

Structure parameters

RF_{obs}	wRF_{obs}	RF_{all}	wRF_{all}	Total Number of reflections	Number of observed reflections (I>3sigma(I))	Number of parameters	Nall/Np
4.71	4.01	5.21	4.15	221	206	18	12.28
